# Relative Humidity Sensors Based on Microfiber Knot Resonators—A Review

**DOI:** 10.3390/s19235196

**Published:** 2019-11-27

**Authors:** Young-Geun Han

**Affiliations:** 1 Gimhae-Harvard Bioimaging Center, Gimhae Industry Promotion and Biomedical Foundation, Gimhae 50969, Korea; yghan@hanyang.ac.kr; 2 Department of Physics, Hanyang University, Seoul 04763, Korea

**Keywords:** fiber-optic sensor, microfiber, microfiber knot resonator, fiber-optic sensor, relative humidity sensor, gas sensor, sensitive materials

## Abstract

Recent research and development progress of relative humidity sensors using microfiber knot resonators (MKRs) are reviewed by considering the physical parameters of the MKR and coating materials sensitive to improve the relative humidity sensitivity. The fabrication method of the MKR based on silica or polymer is briefly described. The many advantages of the MKR such as strong evanescent field, a high Q-factor, compact size, and high sensitivity can provide a great diversity of sensing applications. The relative humidity sensitivity of the MKR is enhanced by concerning the physical parameters of the MKR, including the waist or knot diameter, sensitive materials, and Vernier effect. Many techniques for depositing the sensitive materials on the MKR surface are discussed. The adsorption effects of water vapor molecules on variations in the resonant wavelength and the transmission output of the MKR are described regarding the materials sensitive to relative humidity. The sensing performance of the MKR-based relative humidity sensors is discussed, including sensitivity, resolution, and response time.

## 1. Introduction

Over the past few decades fiber-optic sensors have been intensively deployed in mechanical, chemical, and biological measurement because of their many advantages, including electromagnetic immunity, high accuracy, high sensitivity and flexibility, compactness, and low fabrication cost [1]. In spite of the feasible applications to fiber-optic sensors, controversy continues in terms of commercialization, for example, their small portion of the total sensor market. There is no doubt, however, about the great potential of fiber-optic sensor for future technologies. Recently, microfibers with strong evanescent field to enhance the sensitivity of fiber-optic sensors to external perturbations have been attracting growing interest [2,3,4,5,6,7,8]. The diameter of microfiber is usually in a range from hundreds nanometers to several micrometers [2,3,4,5,6,7,8]. High contrast of refractive index between the microfiber and the environment, diameter uniformity, and sidewall smoothness of the microfiber are attributed to the reduced optical loss, high field confinement, and high sensitivity regarding the strong fractional evanescent field [1,2,3,4,5,6,7,8].

The microfiber knot resonator (MKR) is readily fabricated by making a tie with a microfiber and has specific advantages, including high stability and easy fabrication, reliable response, and high Q-factors regarding effective mode coupling in the intertwisted overlap of the MKR [1,4,5,6,7]. Since light propagating and circulating in the MKR may induce a phase shift multiple of 2π, the periodic optical resonance in the MKR is generated essentially [1]. Since the MKR has good capability to improve the light-matter interaction, it is possible to realize all optical tunable devices based on the MKRs with the nonlinear material overlays [9,10,11,12,13]. In addition, the MKR-based optical sensing techniques have been intensively researched for the measurement of various physical parameters, including temperature, strain, pressure, and refractive index [14,15,16,17,18,19,20,21,22]. Table 1 summarizes various applications of the MKR-based sensing probes and their performance, including the physical parameters of the MKR, sensitivity, resolution, response time, and sensitive materials to enhance the sensitivity of the MKR to external perturbations. A temperature sensor with high sensitivity using the polydimethylsiloxane (PDMS)-packaged MKR was reported [15,16]. High thermal coefficients of the PDMS, including thermo-optic and thermal expansion factors further increased the wavelength shift of the MKR with variations in temperature [15,16]. The mechanical transverse load sensor using the PDMS-encapsulated MKR was proposed [17]. The Young’s modulus of the PDMS can be used to change the knot diameter or length of the MKR with variations in the lateral load resulting in the wavelength shift of the MKR and the realization of MKR-based transverse load sensor [17]. Rather than the waist diameter, the knot diameter has a critical influence on the load sensitivity [17]. The MKR embedded in the steel blade was proposed as the bending sensor because the circular path-length and the effective index of the MKR are easily modified by changing the bending curvature [18]. A magnetic field sensor can be fabricated by inserting the MKR into a glass cell with a magnetic fluid [19]. The external magnetic field alters the transmission characteristics of the MKR depending on its knot diameter [19]. The knot diameter of the MKR regarding the structural bending-induced evanescent field is a significant factor for improving the magnetic field sensitivity of the MKR [19]. With the assistance of a NaCl solution, the MKR without any coating material was exploited to measure the increment of salinity [20]. The hydrogen gas sensor can be fabricated by depositing palladium on the MKR [21,22]. The formation of palladium hydride resulting from the adsorption of hydrogen molecules on the palladium may induce strain regarding the expansion of the palladium in the MKR and the red shift of the wavelength was observed [21,22]. Decreasing the waist diameter improves the hydrogen sensitivity of the MKR [22]. The MKR was also applied to measure electric current around the copper wire [23]. The resonant wavelength of the MKR has red-shift because of the thermal phase-shift induced by the electric current [23].

Among a variety of sensing applications of the MKR, this manuscript specifically aims to review the recent progress of the MKR-based relative humidity sensors regarding their operating principle, structures, and sensitive materials to improve the relative humidity sensitivity. Humidity is an important sensing property in semiconductor and automotive industries, agriculture, chemical and medical areas [8,24,25,26,27]. Relative humidity sensors in medical field are necessary in various respiratory and sterilizer systems, biological products, etc. [28]. The microfiber fabrication method of a silica- or polymer-based MKR, such as a flame blushing or direct drawing technique, respectively, is briefly introduced. The MKR with a high Q-factor, high stability, simple structure, and small size is very suitable for precise measurement of humidity. The ambient index sensitivity is essential to achieve the MKR-based relative humidity sensor. The supplementary techniques to stabilize and enhance the performance and the external index sensitivity of the MKR are described, including Vernier effect or the waist diameter, which is related with the effective group index difference between optical modes. The silica- or polymer-based MKR responds to variation of relative humidity, but with somewhat low sensitivity. The sensitive materials to additionally increase the relative humidity sensitivity of the MKR are discussed, including the deposition method and procedure of sensitive materials, experimental results, and optical phenomena. Various MKR-based relative humidity sensors are compared regarding the physical parameters of the MKR, coating materials, and sensor specifications like sensitivity, resolution, and response time.

## 2. Fabrication, Operating Principle, and Supplementary Method for Improving the Ambient Index Sensitivity of the MKR

**Figure 1:**
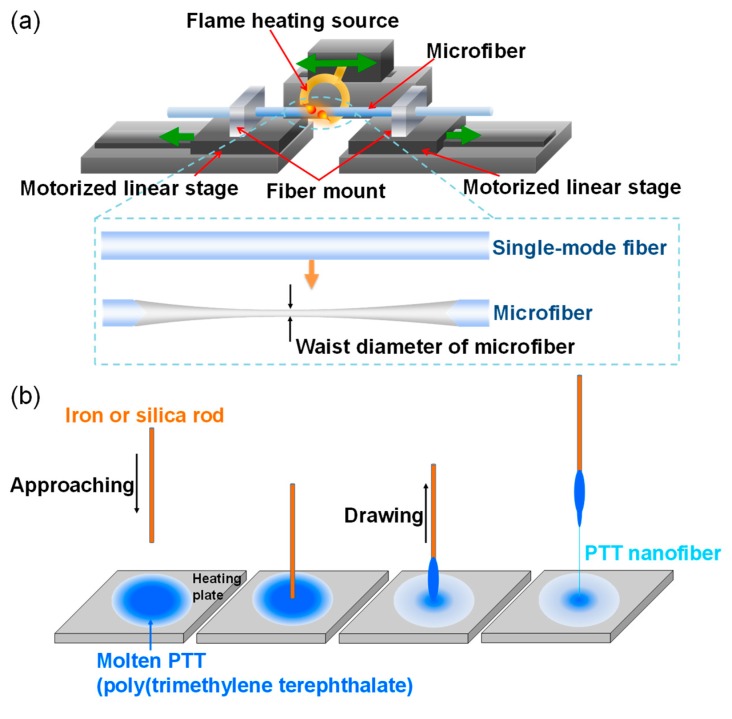
Fabrication of a microfiber [29,30,32]. (**a**) Flame blushing technique for fabrication of a silica-based microfiber [29,30]. Copyright 2016, IEEE; (**b**) direct drawing process for fabrication of a polymer-based micro/nanofiber (polytrimethylene terephthalate, PTT) [31].

The key component of the MKR is a microfiber fabricated by using a micro-tapering technique with various heating sources, including a flame, a laser-based heating tube, and electric strip heater [2,3,4,5,6,29,30]. Two critical parameters in the micro-tapering technique are the temperature of the heating process and elongation of the pulling process. A computer-controlled heater using a flame generates high temperature to soften and melt a single-mode fiber (SMF) which is elongated simultaneously by two motorized stages as shown in Figure 1a. The flow rate of gas like oxygen or hydrogen and the pulling speeds of two motorized stages must be precisely controlled to produce the adiabatic or the non-adiabatic tapered structure of the microfiber. The use of a stereo optical microscope in situ enables observation of variations in the waist diameter. For the polymer optical fiber, a direct drawing method is usually employed because of its simple process as shown in Figure 1b [31]. After melting the polymer on the heating plate, the end of a silica or iron rod is immersed within the molten polymer on the heating plate, resulting in conglutination of polymer. The rod with polymer is then pulled to extend the polymer and produces polymer the micro/nanofiber, as seen in Figure 1b [31].

**Figure 2:**
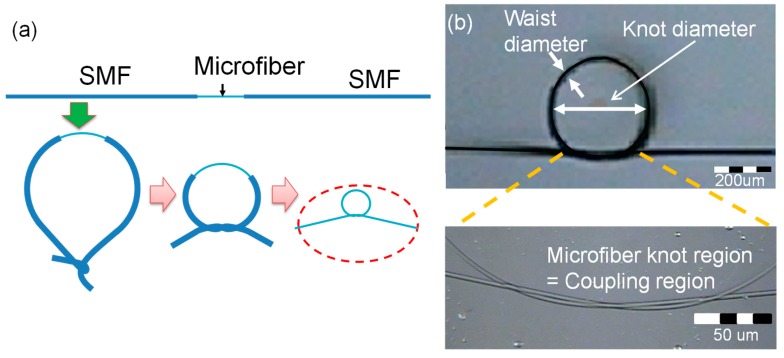
Fabrication of the MKR [29,30]; (**a**) knot procedure of the MKR; (**b**) microscopic image of the MKR. Copyright 2016, IEEE.

**Figure 3:**
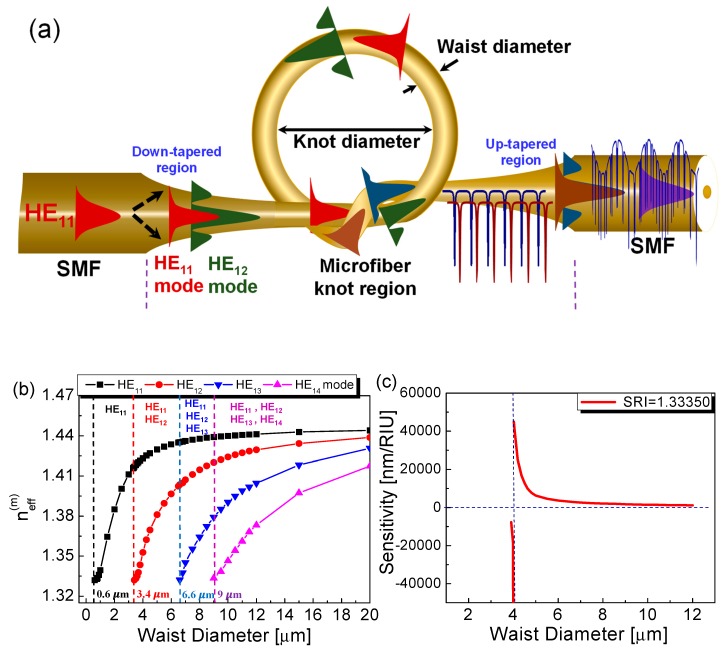
FM-MKR with the improved ambient index sensitivity [29]. (**a**) Operation principle of FM-MKR; (**b**) effective refractive indices (*n*_eff_^(*m*)^) with variations in the waist diameter depending on the radial mode number; (**c**) ambient index sensitivities with variations in the waist diameter (RIU: Relative index unit, SRI: Surrounding refractive index). Copyright 2018, IEEE.

After fabricating a silica or polymer-based microfiber, its two freestanding ends are assembled to form a comparatively large knot and tightened into the desired knot diameter by progressively pulling the free ends of the microfiber, as shown in Figure 2a, so that an MKR with a certain knot diameter can be achieved as shown in Figure 2b [1,29,30]. Mode coupling and interference occur in the microfiber knot region (coupling region) where the microfiber is twisted. Controlling the waist diameter regarding the effective group index difference between two modes (*HE*_11_ and *HE*_12_) is capable of improving the sensitivity of the MKR to external perturbation as shown in Figure 3a–c [29,31]. The waist diameter regarding the V-parameter predominantly determines the number of guided modes regarding the cut-off wavelength and the effective indices of modes as shown in Figure 3b [29]. Figure 3a,b show that few modes are excited in the non-adiabatic down-tapered region when the waist diameter of the microfiber is larger than ~4 μm [29]. Then the few-mode MKR (FM-MKR) can be achieved by making a tie with the few-mode microfiber, which has two optical phenomena regarding optical modal interference in the few-mode microfiber and optical coupling in the FM-MKR. In the FM-MKR, the envelope shape in the transmission spectrum is generated by the modal interference between the *HE*_11_ and *HE*_12_ and the comb-like spectrum is created by optical coupling within the FM-MKR [29]. The sensitivity of the few-mode microfiber modal interferometer to ambient index regarding the waist diameter of the microfiber can be written as [29,32]:

(1) $$S=\frac{\partial \lambda }{\partial n_{amb}}=\frac{\lambda }{\Delta n_{g}^{\left(m\right)}}\frac{\partial \left(n_{HE11}-n_{HE1m}\right)}{\partial n_{amb}}$$

(2) $$\Delta n_{g}^{\left(m\right)}=\Delta n_{\mathrm{eff}}^{\left(m\right)}-\lambda \frac{\partial \Delta n_{\mathrm{eff}}^{\left(m\right)}}{\partial \lambda }$$

where *n_HE_*_11_ and *n_HE_*_1*m*_ are the effective indices of *HE*_11_ and *HE*_1*m*_ modes, respectively. *m* is the mode order and Δ*n_g_*^(*m*)^ is the difference in effective refractive group indices between the *HE*_11_ and *HE*_1*m*_ modes. The effective indices of the two modes (*n_HE_*_11_ and *n_HE_*_1*m*_) and the value of Δ*n_g_*^(*m*)^ can be changed by the waist diameter of the few-mode microfiber [29,32]. Equation (1) shows that the ambient index sensitivity can be improved by reducing Δ*n_g_*^(*m*)^ regarding the waist diameter of the few-mode microfiber. Figure 3c exhibits the theoretical result on the ambient index sensitivity of the few-mode microfiber modal interferometer with respect to the waist diameter. Since Δ*n_g_*^(*m*)^ becomes zero at 4-μm waist diameter, the ambient index sensitivity (*S*) is significantly enhanced, as shown in Figure 3c [29].

**Figure 4:**
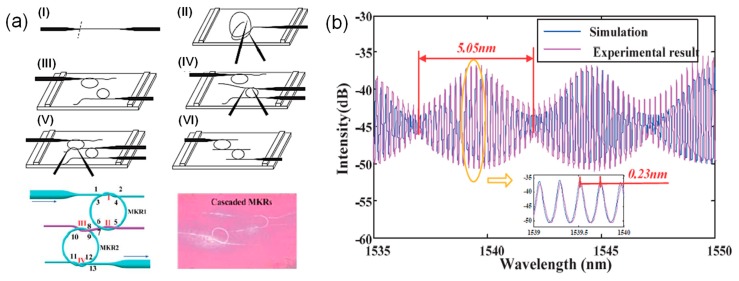
Cascaded MKRs with Vernier effect [33]. (**a**) Fabrication procedure, configuration, and photograph of the cascaded MKRs; (**b**) transmission spectrum. Theoretical analysis, including all mathematical models and parameters was described in [33] in detail. [Reprinted/Adapted] with permission from [33] © The Optical Society.

By cascading two MKRs with slightly different free spectral ranges (FSRs), the Vernier effect based on optical spectrum interrogation can be induced to improve the ambient index sensitivity of the MKR [33]. After fabricating three pieces of microfibers, two MKRs were fulfilled by making ties with two microfibers as shown in Figure 4a ((I)–(III)). The other microfiber, in sequence, is carefully knotted with two MKRs in series, as shown in Figure 4a ((IV)–(VI)), to form the cascaded MKRs [33]. The output spectrum of the concatenated MKRs is created by the product of the individual transmission spectrum of the MKR, as seen in Figure 4b. Therefore, peak wavelengths of the cascaded MKRs are observed in the transmission spectrum such that two interference peaks are overlapped fractionally. The wavelength shift of the cascaded MKRs with variations in external perturbation can be increased by a magnification factor (*M* = *FSR*_1_/|*FSR*_1_ − *FSR*_2_|) [33].

## 3. Relative Humidity Sensors Based on MKRs with Sensitive Materials

**Figure 5:**
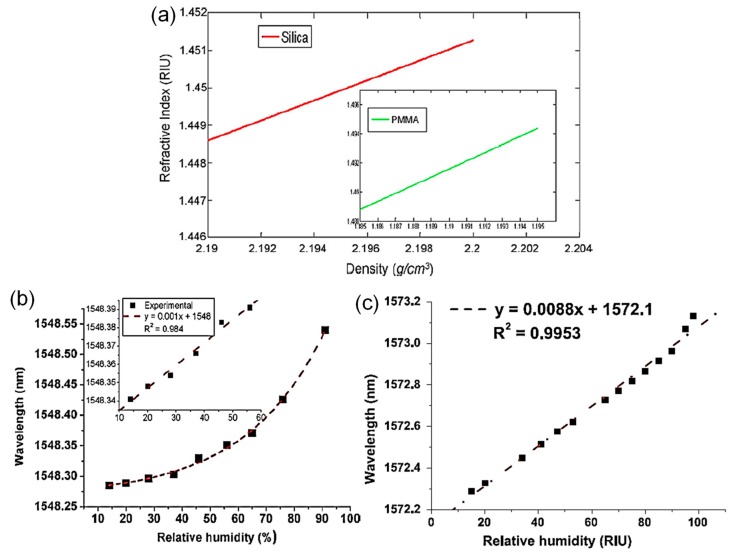
Silica or polymer MKR relative humidity sensor [34]. (**a**) Refractive index of silica or polymer (polymethyl methacrylate, PMMA) with variations in density; wavelength shift of the silica (**b**) or the polymer MKR (**c**) as a function of relative humidity. Copyright 2011, Elsevier.

A silica- or polymer-based MKR without any humidity-sensitive material was applied to measure relative humidity in the surrounding environment [34]. A chamber with a hygrometer at a constant temperature is usually deployed in the experiment for relative humidity measurement. In the chamber, relative humidity is controlled by the amount of wet or dried air. Since the refractive index of silica or polymer is affected by relative humidity, the wavelength of the MKR is shifted. The density of the silica or polymer is changed by absorbing water vapor molecules so that refractive index is varied regarding the expansion or contract of the medium, as seen in Figure 5a [34]. Swelling the medium after adsorbing water molecules mitigates the density and the medium refractive index. Shrinking the medium after absorbing water vapor molecules increases the density and refractive index of medium because water vapor molecules fill the interstitial gaps of the medium [34]. For the silica-based MKR, increasing relative humidity shifts the wavelength into longer wavelengths [34]. This may be explained by the porous matrix of the silicon trapping water molecules on its interior surface [34]. Since water vapor molecules infiltrate into the porous matrix of the silicon, the average density of silicon regarding its refractive index is increased [34]. Consequently, a red-shift of the wavelength of the silica-based MKR was observed as shown in Figure 5b [34]. In a range of relative humidity from 20% to 60%, the linear behavior of the resonant wavelength shift regarding relative humidity was observed as shown in the inset of Figure 5b because of the variation of average density of the silicon resulting in the increase of its refractive index [34]. At a high relative humidity of more than 60%, the wavelength shift increased rapidly, which is attributed to the formation of clusters and the aggregation of water molecules on the silica-based MKR as shown in Figure 5b [34]. For the polymer-based MKR, as seen in Figure 5c, the peak wavelength also shifts to longer wavelengths and its relative humidity sensitivity is higher than that of the silica-based MKR because of the high hydrophilic feature of the polymer regarding the large molecule and the gap between molecules in the polymer [34]. At a high relative humidity of more than 90%, the amount of resonant wavelength shift increased because of the enhanced molecular state adsorption of water on the polymer surface [34].

**Figure 6:**
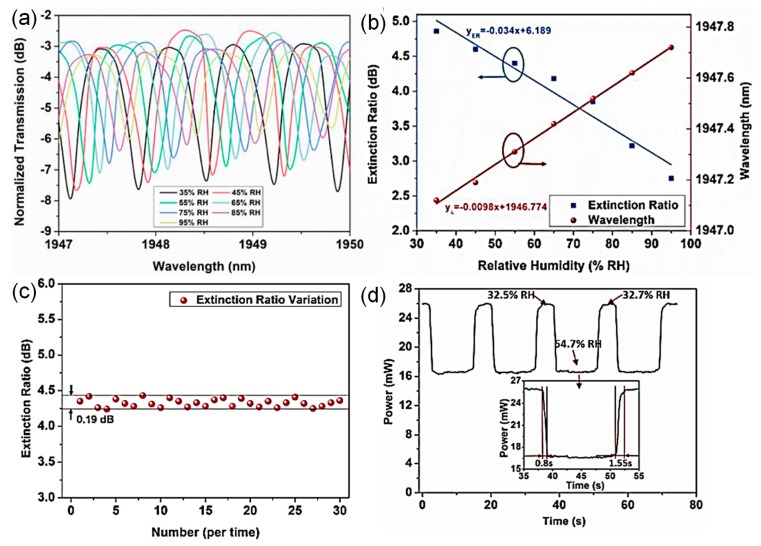
2-μm waveband MKR relative humidity sensor [35]. (**a**) Normalized transmission spectra with variations in relative humidity; (**b**) variations of extinction ratio and peak wavelength as functions of relative humidity; (**c**) fluctuation of extinction ratio for 30 consecutive measurements at a relative humidity level of 57%; (**d**) temporal response. Copyright 2019, IEEE.

The MKR-based relative humidity sensor at 2-μm waveband was proposed [35]. Since the strong water absorption exists at a wavelength of 1950 nm, the detection efficiency of relative humidity using the MKR can be improved without additional coating materials [35]. A tunable laser (OETLS-300) in a wavelength range from 1950 nm to 2050 nm was utilized as an input light source. An InGaAs photodetector was synchronized with a tunable laser by using a computer to recognize the wavelength shift of the MKR-based sensing probe [35]. The output spectrum of the 2-μm waveband MKR was changed by increasing relative humidity as depicted in Figure 6a [35]. Adsorption of water vapor molecules on the 2-μm MKR increases its refractive index, resulting in the red shift of wavelength, as shown in Figure 6b. Light absorption at 1950 nm reduces the extinction ratio of the MKR with increases in relative humidity. In Figure 6c, the fluctuation of the extinction ratio was approximately 0.19 dB for 30 consecutive measurements at a relative humidity of 57%. The rising and falling times were ~0.8 and ~1.55 s, respectively, as shown in Figure 6d [35].

**Figure 7:**
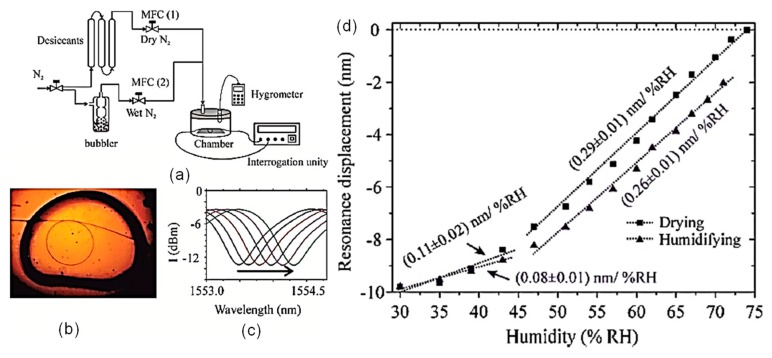
Nafion-deposited MKR relative humidity sensor [36]. (**a**) Experimental scheme for measurement of relative humidity using the MKR with Nafion; (**b**) photograph of the MKR deposited by Nafion; (**c**) transmission spectra with variations in relative humidity; (**d**) wavelength shift as a function of relative humidity. The system humidity calibration curve is shown in the inset. [Reprinted/Adapted] with permission from [36] © The Optical Society.

A supplementary coating layer as a receptor or transducer is exploited to apply the MKR to a relative humidity sensor [36]. The MKR with the strong evanescent field has an inherently high sensitivity to surrounding index change, so that implementing a humidity-sensitive material is effective for transforming the ambient index sensitivity to the relative humidity one. Nafion with high hydrophilicity, low refractive index, and high adhesivity to silica was exploited to realize the MKR-based relative humidity sensor [36]. A 3 μL of a 5 wt. % Nafion solution (in low alcohols and 10% water) was manually dropped on the MKR and then dried for 24 h at room temperature as shown in Figure 7a,b [36]. The Nafion-deposited MKR was placed in a chamber with a hygrometer. The relative humidity within the chamber was controlled by a gas blender. Wet air was provided by bubbling it into bubbling flasks containing distilled water. Dry air was obtained by passing an air flow through desiccant columns of silica gel and molecular sieve 5A (Sigma–Aldrich) [36]. Nafion as a perfluorosulfonate ion exchange polymer was developed by DuPont and has been extensively utilized in fuel cells [37] and electrochemical/optical sensors [36,38,39,40,41]. Nafion thin film has interesting properties, such as increasing relative humidity swells its structural dimension and reduces its refractive index [36,38,39,40,41]. The effect of relative humidity on the mechanical stress of the Nafion thin film predominantly induces a red shift of wavelength, as depicted in Figure 7c,d [36]. Two distinguished sensitive humidity regions are apparently observed in Figure 7d. At lower-mid relative humidity (30~45%), two relative humidity sensitivities (0.11 ± 0.02 nm/% for humidity increase and 0.08 ± 0.01 nm/% for humidity decrease) were exhibited. At higher-mid relative humidity (45~75% RH), two different sensitivities, such as 0.29 ± 0.01 nm/% for humidifying increase and 0.26 ± 0.01 nm/% for dehumidifying, are observed. A sensing hysteresis of ~1.9 nm was measured during the measurement. This is probably explained by the fact that the swelling tension of the Nafion thin film regarding the absorption of relative humidity degrades the sensitivity of relative humidity and induces hysteresis and two different sensitivities depending on the relative humidity ranges [36].

**Figure 8:**
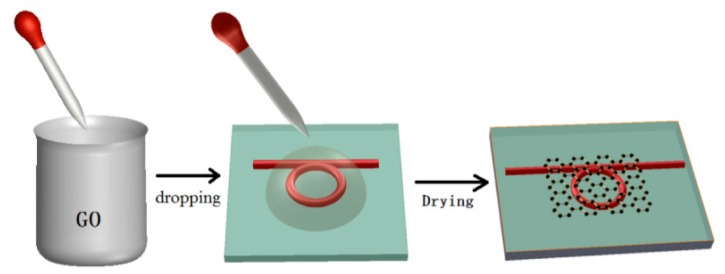
Coating process of graphene oxide on the MKR [43]. Reprinted/adapted with permission from The Optical Society ©.

**Figure 9:**
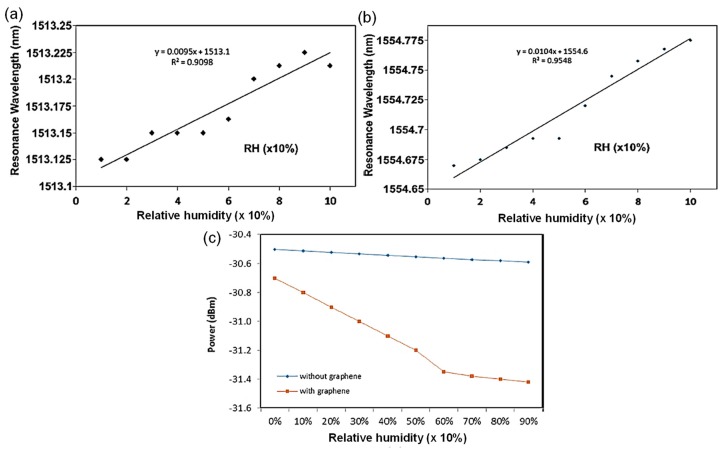
Graphene oxide-based MKR relative humidity sensor [42]. Wavelength shifts of MKRs without (**a**) and with (**b**) graphene oxide as a function of relative humidity; (**c**) output power variations of MKRs without and with graphene oxide as a function of relative humidity. Copyright 2018, Elsevier.

Two-dimensional materials, including graphene and reduced graphene oxide, were utilized as sensitive materials to ameliorate the relative humidity sensitivity of the MKR [42]. Graphene or reduced graphene oxide is suitable for gas- or biomolecule adsorption with large surface area, strong affinity, and high adsorption [42,43,44,45,46,47]. After dissolving the graphene oxide in deionized water, the graphene oxide solution was sonicated in an ultrasonic bath for 30 min [43]. The graphene oxide, in sequence, was dropped on the MKR fixed on the MgF_2_ substrate and dried at a temperature of 40 °C as shown in Figure 8 [43]. For the MKR without the graphene oxide, variations of refractive index of the air regarding relative humidity alter the extinction ratio, output power, and the resonant wavelength, which are less distinct. Additional sensitive materials further improve the relative humidity sensitivity of the MKR. Since the graphene oxide layer has the high level of hygroscopicity, water molecules are absorbed by the graphene oxide matrix [42]. Therefore, the graphene oxide coating significantly develops the relative humidity sensitivity of the MKR [42]. The resonant wavelength of the MKR without or with the graphene oxide coating shifts into longer wavelengths as relative humidity increases as shown in Figure 9a,b. The MKR with the graphene oxide overlay has higher relative humidity sensitivity than the ordinary MKR [42]. The output power in the interference spectrum of the MKR without a graphene is barely reduced, as seen in Figure 9c. For the MKR with the graphene overlay, however, the output power is rapidly dropped from −30.7 dBm at a relative humidity of 0% to −31.3 dBm at a relative humidity of 60% as shown in Figure 9c [42]. As the relative humidity value is further increased to be 90%, the output power is slowly mitigated and dropped to −31.4 dBm because of the absorption saturation of water molecules in the graphene oxide layer as shown in Figure 9c [42]. The use of the graphene oxide overlay on the MKR successfully improves the sensitivity and linearity to relative humidity change.

**Figure 10:**
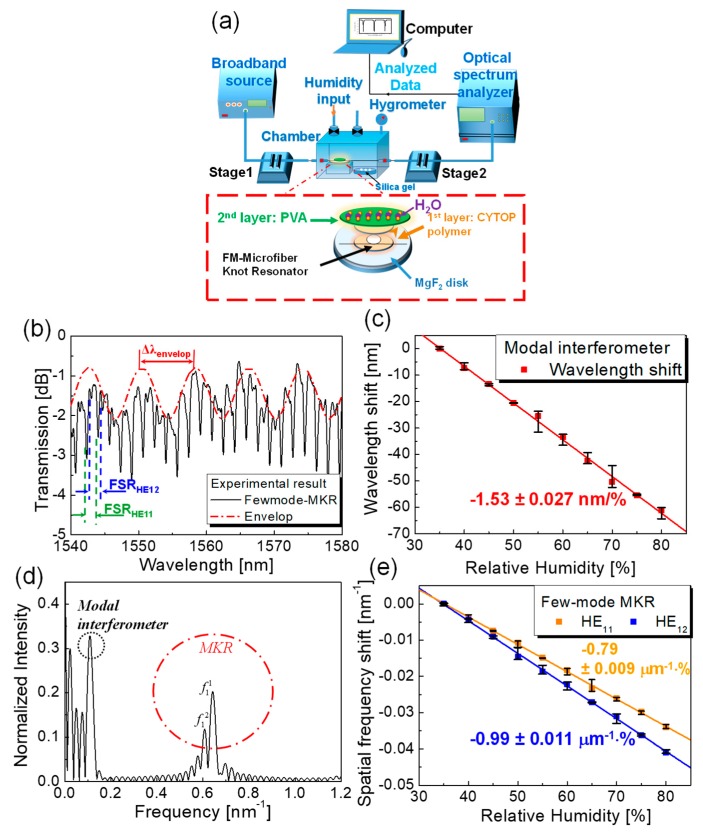
PVA-based FM-MKR relative humidity sensor [29]. (**a**) Experimental configuration; (**b**) transmission spectrum; (**c**) resonant wavelength shifts with variations in relative humidity; (**d**) spatial frequency spectrum; (**e**) spatial frequency shifts with variations in relative humidity. Copyright 2018, IEEE.

A polyvinyl alcohol (PVA) polymer was employed as an absorber of relative humidity to realize a MKR-based relative humidity sensor [29,30,48,49,50,51,52,53,54]. FM-MKR with two modes (*HE*_11_ and *HE*_12_) was fabricated by using a flame-blushing method [29]. The non-adiabatic tapered shape in the microfiber is able to excite asymmetric modes (*HE*_1*m*_) from a fundamental symmetric mode (*HE*_11_) regarding the waist diameter [29]. The few-mode MKR on a low index MgF_2_ disk was spin-coated by 9%-Cytop (n_Cytop_ = 1.34) at 700 rpm for 60 s and annealed in sequence for 120 min at 80 °C. Then the 8% PVA overlay with a thickness of 1.3 μm was spin-coated at 1200 rpm for 30 s and annealed again for 40 min at 60 °C and continuously for 180 min at 80 °C as shown in Figure 10a [29,30]. Figure 10b shows that the transmission of the FM-MKR was optically characterized by superimposing the spectrum of the modal interferometer with that of the MKR [29]. In the few-mode MKR, the envelope shape in the transmission spectrum is generated by the modal interference between the *HE*_11_ and *HE*_12_ regarding the slow-varying envelop in the transmission. Optical coupling within the few-mode MKR creates the comb-like spectrum regarding the fast-varying transmission as shown in Figure 10b. Since PVA is a water-soluble compound, absorbing water molecules swells the PVA overlay, which reduces its density. Consequently, the refractive index of the PVA is diminished by increasing relative humidity in the surrounding environment, which, in turn, changes the output spectrum of the MKR. Figure 10c shows that ascending the concentration of relative humidity shifts the resonant wavelength into shorter wavelengths [29]. Optimizing the waist diameter regarding the group index difference between the two modes improves the relative humidity sensitivity of the FM-MKR sensor [29]. Figure 10d exhibits the spatial frequency spectrum after converting the optical spectrum of the MKR with the fast Fourier transform. In Figure 10e, it is noticeable that all spatial frequencies shift to lower ones by increasing relative humidity because of the effective index reduction of the two modes.

Titanium dioxide (TiO_2_) nanoparticles were utilized to attain the MKR-based relative humidity sensor [55,56,57,58,59]. The TiO_2_ nanoparticle-based overlay has fast adsorption capability for water vapor molecules because of its porous structure at room temperature [56,57,58,59]. The anatase phase of TiO_2_ nanoparticles has better water adsorption ability than the two other phases, rutile and brookite [55]. The commercially available anatase TiO_2_ powder was immersed with deionized water in a glass bottle [55,56,57,58,59]. The solution with TiO_2_ nanoparticles was homogenized by the ultrasonication process and coated immediately on the surface of the MKR [55]. TiO_2_ nanoparticles can be readily deposited on the MKR because of the evaporation of DI water, light injection, and the optical tweezing effects regarding the strong evanescent field of the MKR [55]. The output power of the MKR was measured to confirm the attachment of TiO_2_ nanoparticles on the MKR. The transmission power of the MKR decreases monotonically as TiO_2_ nanoparticles begin to be deposited on the surface of the MKR after immersing the MKR in the TiO_2_ solution [55]. The transmission power is abruptly changed after taking the TiO_2_ nanoparticle-coated MKR out of the TiO_2_ solution and stabilized within a few seconds [55]. Increasing relative humidity shifts the wavelength of the TiO_2_^−^ coated MKR into longer wavelengths because of the refractive index increment of the TiO_2_ regarding the water filling process. The extinction ratio is reduced by increasing relative humidity. The deposition of TiO_2_ nanoparticles on the MKR is effectively capable of improving the relative humidity sensitivity. The fast response and slow recovery time of the MKR with the TiO_2_ coating are probably caused by the fast diffusion and slow desorption of water molecules regarding TiO_2_ nanoparticles [55].

## 4. Conclusions

As presented, it is manifest that the MKR has great potential in various sensing applications to environmental, biomedical, and chemical monitoring. A great diversity of the MKR-based sensing probes with different sensitive materials has been researched. MKR is structurally based on microfiber. The fabrication methods of the silica- or polymer-based microfibers, including a flame blushing or direct drawing technique, respectively, were first presented. The MKR was simply realized by making a knot with a microfiber. The strong evanescent field and high Q-factor of the MKR enable the provision of various sensing capabilities to monitor physical, mechanical, and chemical parameters. The great adaptability of various coating materials and nanoparticles to the MKR facilitates the development of its sensitivity to external perturbation change and the extension of the MKR-based sensing capabilities. The applications and performance of the MKR-based sensors are summarized in Table 1. A variety of research and development of the MKR-based relative humidity sensors was overviewed regarding the physical properties of the MKR to improve the relative humidity sensitivity, sensitive materials to relative humidity, and the sensing performance like sensitivity, resolution, and response time. Adsorption of the water vapor molecules on the silica- or polymer-based MKR or the supplement coating materials may change the refractive index and the mechanical stress (swelling or shrinking) of materials and consequently modify the transmission characteristics of the MKR regarding the resonant wavelength and the output power. The performance of the MKR-based relative humidity sensors, including sensitivity, resolution, and response time is summarized in Table 2. Although the coating materials can effectively improve the relative humidity sensitivity of the MKR, they degrade the response time of the MKR-based sensor.

The small size of the MKR is advantageous for the integration and compatibility with fiber-optic sensors. We emphasize the need for additional research on the MKR-based relative humidity sensor focusing on practical and robust package techniques, stability and reproducibility, discrimination of multiple physical parameters, sensing signal interrogation, massive production, and automation. The micro/nano fabrication technology needs to be developed to stabilize all procedures involved in the MKR fabrication and the deposition of the sensitive materials. New materials sensitive to relative humidity change should be investigated to improve the response time of the MKR and the long-term stability. New configurations of the MKR-based humidity sensor incorporating new sensitive materials, new types of optical fibers (e.g., multicore fibers, few-mode fiber), and other fiber-optic sensors will be exported.

## Figures and Tables

**Table 1:** Performance comparison of the MKR-based sensors.

Authors/Publication Year	Sensor Type	Waist Diameter of Microfiber	Knot Diameter of MKR	Sensitive Material	Sensitivity	Resolution/Accuracy	Response Time
					Wavelength		
J. Li et al./ 2017 [15]	Temperature	Not Available	4.5 mm	Poly- Dimethylsiloxane (PDMS)	1.408 nm/°C for 24–38 °C 0.973 nm/°C for 40–54 °C	0.014 °C	Not Available
R. Fan et al./ 2019 [16]	Temperature	8.6 μm	4.5 mm	w/o Poly- Dimethylsiloxane (PDMS)	183 pm/°C w/o PDMS	Not Available	Not Available
				Poly- Dimethylsiloxane (PDMS)	1.67 nm/°C w PDMS		15 s
J. Li et al./ 2018 [17]	Load	6.92 μm	6.0 mm	w/o Poly- Dimethylsiloxane (PDMS)	6 pm/N w/o PDMS	Not Available	Not Available
		7.2 μm	4.0 mm	Poly- Dimethylsiloxane (PDMS)	90 pm/N w PDMS	0.38 N	Not Available
S. Dass et al./ 2018 [18]	Bending	16 μm	885 μm	None	3.04 nm/m^−1^	3.29 × 10^−3^ m^−1^	Not Available
Y. Ly et al./ 2018 [19]	Magnetic Field	4.0 μm	155 μm	Water-Based Magnetic Fluid	277 pm/mT	0.07 mT (Accuracy)	Not Available
Y. Liao et al./ 2015 [20]	Salinity	2.5 μm	855 μm	None	21.18 pm/%o	Not Available	Not Available
X. Wu et al./ 2015 [22]	Hydrogen	2.98 μm	7.25 mm	Palladium (Pd)	Not Available	Not Available	Not Available
K. S. Lim et al./ 2011 [23]	Current	2.0 μm	185 μm	None	51.3 pm/A^2^	Not Available	3 s

**Table 2:** Performance comparison of MKR-based relative humidity sensors with different sensitive materials and the physical parameters of MKRs.

Authors/Publication Year	MKR Material	Waist Diameter of Microfiber	Knot Diameter of MKR	Humidity-Sensitive Material	Sensitivity		Resolution (*RH: Relative Humidity)	Response Time
					Wavelength	Transmission Power		
Y. Wu et al./2011 [34]	Silica MKR	1.2 μm	500 μm	Not Available	~12 pm/10% RH	Not Available	0.017% RH	Not Available
	Polymer MKR	2.1 μm	500 μm	Not Available	~88 pm/10% RH	Not Available	0.0023% RH	~0.5 s
K. Xu et al./2019 [35]	Silica MKR at 2-mm Waveband	1 μm	395 μm	Not Available	~10 pm/% RH	0.034 dB/% RH	Not Available	Rising: ~0.8 s Falling: ~1.55 s
M. Gouveia/2014 [36]	Silica MKR	3 μm	250 μm	Nafion	0.11 nm/% RH for low RH 0.29 nm/% RH for High RH	Not Available	Not Available	Not Available
S. Azzuhria et al./2018 [42]	Silica MKR	Not Available	3.02 μm	Graphene Oxide	0.0104 nm/% RH (0.0095 nm/% w/o Graphene Oxide)	Not Available	0.096 % RH (0.1% RH w/o graphene oxide)	Not Available
A. D. D. Le et al./2018 [29]	Silica MKR	4 μm	380 μm	Polyvinyl Alcohol (PVA)	-1.53 nm/% RH	Not Available	Not Available	Not Available
M. Faruki et al./2016 [55]	Silica MKR	2.1 μm	2.5 mm	Titanium Dioxide (TiO_2_)	2.5 pm/% RH (1.3 pm/%RH w/o TiO_2_)	0.0836 dB/% RH (0.0626 dB/%RH w/o TiO_2_)	Not Available	Response: ~25 s Recovery: ~30 s
